# Mapping the journey of families navigating problem drinking in South Asia: a scoping review

**DOI:** 10.1186/s12889-025-22967-y

**Published:** 2025-05-09

**Authors:** Nikitha Sibil Rebello, Varalakshmi Chandra Sekaran, Lena Ashok, Ajay Bailey, Deepak Mallya, Divya Sussana Patil

**Affiliations:** 1 Department of Global Public Health Policy and Governance, Prasanna School of Public Health, Manipal Academy of Higher Education, Manipal, Karnataka India; 2 Department of Social and Health Innovation, Prasanna School of Public Health, Manipal Academy of Higher Education, Manipal, Karnataka India; 3 Department of Human Geography and Spatial Planning, Faculty of Geosciences, Utrecht University, Utrecht, The Netherlands; 4 Transdisciplinary Centre for Qualitative Methods, Department of Data Science, Prasanna School of Public Health, Manipal Academy of Higher Education, Manipal, Karnataka India; 5 Department of Psychiatry, Dr A V Baliga Memorial Hospital, Udupi, Karnataka India; 6 Centre for Evidence-informed Decision-making, Prasanna School of Public Health, Manipal Academy of Higher Education, Manipal, Karnataka India

**Keywords:** Parental problem drinking, Alcohol drinking, Substance use, Alcohol dependence syndrome, Family violence, Intimate partner violence, Adolescent behaviour

## Abstract

**Background:**

Problem drinking is a serious issue as it affects persons with problem drinking and family members including children, adolescents, spouses, and extended family members, thus affecting the entire family environment. This review aims to explore the evidence regarding challenges faced by family members including adolescent children, spouses, and extended family members due to a person with a problem drinking. The findings from the review will help inform further research to develop interventions for family members and for parents having problem drinking issues.

**Methods:**

This scoping review was undertaken using the Joanna Briggs Institute framework. Six online databases were searched for relevant articles published in English from January 2003 to June 2023. Both quantitative and qualitative studies conducted in South Asia on challenges faced by family members living with either parent/s with problem drinking were included. Challenges were sorted into physical, psychological/emotional, social, and financial, as faced by family members.

**Results:**

Forty-seven articles met the inclusion criteria. Of the 47 studies, 37 studies described challenges faced by spouses while 10 studies described challenges among adolescent children in households with parental problem drinking. Most studies focused on psychological/emotional challenges while more than half reported physical challenges. Since the scoping review aimed to map the evidence available on challenges faced by family members of a person with problem drinking, data was presented narratively.

**Conclusions:**

This scoping review explored multifaceted challenges and consequences among families affected by a person with problem drinking. The review has provided insights into the complexities of problem drinking leading to disruptive family relationships, poor parenting, emotional vulnerabilities, and psychiatric morbidities. Healthcare professionals need to consider the challenges that affect the family while also considering each individual in the family in managing parental problem drinking.

**Supplementary Information:**

The online version contains supplementary material available at 10.1186/s12889-025-22967-y.

## Background

Problem drinking is referred to as a family disease since it affects everyone in the family living with the individual who consumes alcohol [1]. It can cause conflict in family cohesion affecting spousal and parent–child relationships [2, 3]. The family functions as the primary source of attachment, interdependence, socialization, and nurturing [4–6]. The functionality of the family is challenged by stressful events such as a family member's illness or death, divorce, strained relationships, employment issues, or hardships including substance use [7–9]. Substance use including problem drinking is a multipronged phenomenon with causes found in the interaction of biopsychological, familial, and social factors [6, 10]. Globally, drinking alcohol contributes to 3 million deaths each year as well as to millions of disabilities and ill health [11]. The Sustainable Development Goals (SDGs) were launched by the United Nations, which are a global effort to eradicate poverty and improve the quality of life of people. The inclusion of substance use, including harmful use of alcohol reflects in the scope of the global developmental agenda [12]. Harmful use is accountable for 5.1% of the global burden of disease [11] with an impact on Sustainable Development Goals (SDGs). The core objectives of the United Nations Agenda 2030 are health and well-being. Addressing mental health needs lines up with multiple SDGs. Alcohol-related harm prevents the achievement of at least 14 of the 17 SDGs [13]. As alcohol causes social and economic harm, Target 3.5 highlights how reducing alcohol consumption and associated harm might help in achieving the SDGs [13]. In addition, alcohol consumption affects the social, economic, and environmental dimensions of SDGs [13]. Due to the possibility of spending a greater percentage of household income on alcohol or the treatment of diseases linked to alcohol use, families affected by problem drinking are more susceptible to hunger and poverty [13]. Problem drinking encompasses a spectrum of alcohol problems from excessive misuse to alcohol use disorder [14]. Problem drinking is a serious issue as it affects the parent with problem drinking and family members including children, adolescents, spouses, and extended family members, thus affecting the entire family environment [15]. The alcohol market is on the rise in the Asian context with sales upward of 30% compared to global rates, growing 176% between 2000 to 2019 [16]. The rise in alcohol consumption is also reflected among South Asian nations resulting in 14.7 million disability-adjusted life years and 340,000 deaths [17]. Per the Sri Lankan Ministry of Health, Nutrition & Indigenous Medicine, current alcohol use was measured at 23.7%. The use of alcohol was higher among males at 48.1% as compared to females which was significantly lower at 1.2% [18]. In neighbouring India, it is projected that by 2050, alcohol consumption alone will result in the loss of 552 million quality-adjusted life years and a net economic loss of INR 97.9 lakh crore, or 1.45% of the country's GDP [19]. Comparatively, Bangladesh reported low alcohol use at 2% over the past year. In contrast, among current drinkers, 77% were binge drinkers contributing to a public health concern [20]. Dewan and Chowdhury [21] reported 20.2% indulging in heavy episodic drinking in Bangladesh and families with a prior history of drinking contributed to the prevalence. Prior research on problem drinking has focused on consequences surrounding the family [22–27] albeit largely in the western context.

Murray Bowen’s family systems theory underpins the theoretical framework of this scoping review exploring the challenges faced within the family and between family members affected by a person with problem drinking. The key concepts of Murray Bowen’s family systems theory include triangles, differentiation of the self, nuclear family emotional process, family projection process, multigenerational transmission process, emotional cutoff, sibling position, interconnectedness, and societal emotional process. The family is, hence, identified as a complex emotional system where members are interconnected, and one influences the other. In these families, problem drinking affects one member which can, in turn affect the rest of the family [28]. This theory explains the processes by which parents’ problem drinking affects the household [29]. Relationships within the family are complex and reciprocal. Family members' behaviors are determined by the transactional patterns within the family system. In times of stress or crisis, the family tries to revert to its initial state in which all its members were comfortable and functional. Any alteration made to one of these members will inevitably affect all the other members and hence the system as a whole.

There is an increasing interest in understanding the consequences of problem drinking on people other than the problem drinker himself [25]. However, a comprehensive review of literature on the effect of problem drinking and its consequences on family members in the South Asian countries is lacking. Such reviews have been carried out in other regions such as Oceania and North American region [30, 31]. Based on our initial literature search, we did not come across a scoping review that mapped the specific challenges faced by families including marital conflict, violence against the spouse and/or children, psychosocial and financial challenges stemming from problem drinking in the South Asian context. Therefore, this scoping review aims to explore the evidence on challenges faced by family members including adolescent children, spouses, and extended family members due to individual with problem drinking. The findings from the review will help inform further research to develop interventions for family members and for parents having problem drinking issues.

## Methods

To map the challenges faced by family members due to person with problem drinking in South Asia we adopted the Arksey & O’Malley framework to conduct the scoping review [32]. The review has been reported according to the Preferred Reporting Items for Systematic Reviews and Meta-analysis extension for scoping review studies (PRISMA-ScR) guidelines [33]. See Additional file 3 for more details. The review protocol was registered on Open Science Framework (OSF), which can be accessed at https://osf.io/dhrxt/. The steps followed are as follows:

### Step 1: defining the review question

The review team used the Population (P), Concept (C), and Context (C) framework to develop the following research question.

*What are the challenges faced by family members due to a person with problem drinking in South Asia?*

The PCC criteria were used to define the inclusion criteria for the review.

Population: We included studies conducted among spouses, adolescents, and extended family members of individuals with problem drinking.

Concept: For this review, we define challenges as “conditions precipitated by an individual with problem drinking that involve difficulties, harms, and the consequences of problem drinking”. We excluded studies that reported problem drinking among other extended family members and adolescents.

Context: We included studies conducted in South Asian countries such as India, Pakistan, Afghanistan, Bangladesh, Bhutan, Maldives, Sri Lanka, and Nepal. Multi-country studies on the topic were included if separate analysis for South Asian countries is available.

Study design: We included both quantitative and qualitative studies conducted in South Asia on challenges faced by family members living with either parent/s with problem drinking. We excluded protocols, reports, editorials, opinion pieces, book chapters, and conference proceedings.

### Step 2: identifying relevant studies

We searched databases such as PubMed (NCBI), Scopus (Elsevier), CINAHL(EBSCO), Web of Science (Clarivate), ProQuest Psychology, and PsycINFO to identify relevant studies in South Asia published between January 2003 and June 2023. Journal articles published in the English language were included. We followed a systematic search strategy initially designed for PubMed, which was adapted for each database. The search was formed using the combination of Boolean operators AND, OR, and NOT with search terminologies. The search engine-specific strategy used in PubMed has been attached in the supplementary data file (Additional file 1).

### Step 3: selection of studies

The studies found in all search engines were uploaded into'Rayyan' [34] an intelligent-systematic review online platform and duplicates were removed. We followed a two-stage screening process, title/abstract and full-text screening. Based on title/abstract screening, we excluded articles that did not fit the population-concept-context-study design. Two researchers independently screened the title and abstracts against the eligibility criteria. Articles reviewed, as included by both reviewers, were considered further for full-text screening. In case of a conflict between the two reviewers in the title/abstract screening, a third reviewer solved the conflict and the third reviewer's opinion for inclusion was considered. Full texts of the articles were retrieved and screened by two reviewers. The option to exclude was discussed by the reviewers until they reached a consensus.

### Step 4: data extraction

Based on the reviewer's clinical experience and knowledge as well as a literature review of the'Population-Concept-Context'study design, we prepared a data extraction file using Google spreadsheet. The data extraction file comprised citation details, country/region, study design, participant description, data collection tool, and challenges faced by family members due to a person with problem drinking. Challenges were listed based on physical, psychological/emotional, social, and financial challenges faced by family members. We did not critically assess the quality of individual sources of evidence used in this scoping review.

### Step 5: data analysis and presentation

Since the scoping review aimed to map the evidence available on challenges faced by family members of individuals with problem drinking, data was presented diagrammatically, which was accompanied by the detailed narrative summary of identified challenges.

## Results

**Fig. 1:**
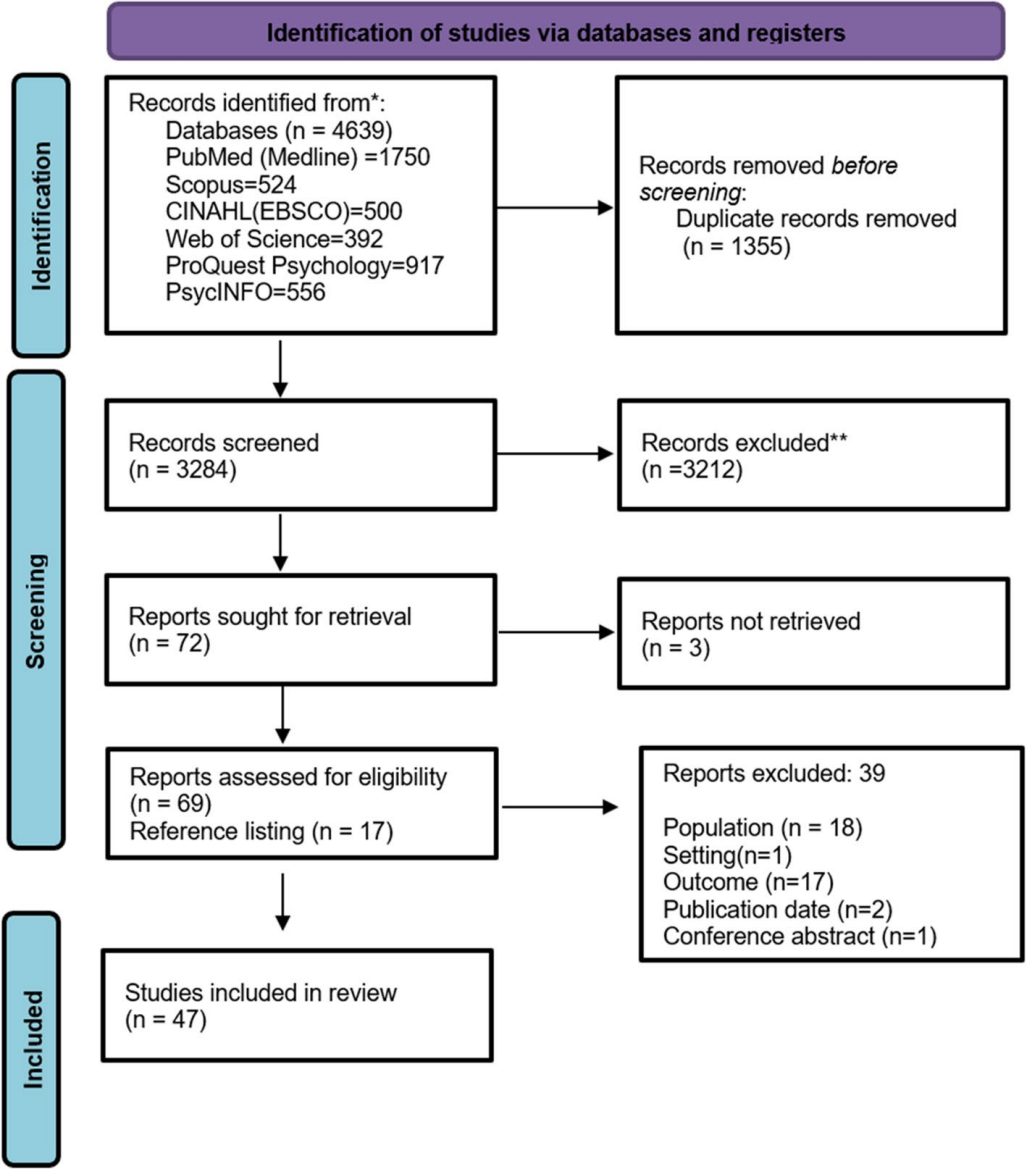
PRISMA 2020 flow diagram for scoping review

Search conducted on electronic databases resulted in 4,639 citation hits. After the removal of duplicates, 3,284 documents were screened for title and abstracts based on the inclusion and exclusion criteria. Following this, 72 full texts were screened, of which 30 studies were included. Further 17 studies were found eligible to be included from the reference list of included studies. Therefore, a total of 47 studies were included for synthesizing and reporting the results. The detailed study selection process has been documented using PRISMA-2020 (Fig. 1).

### Characteristics of included studies

**Fig. 2:**
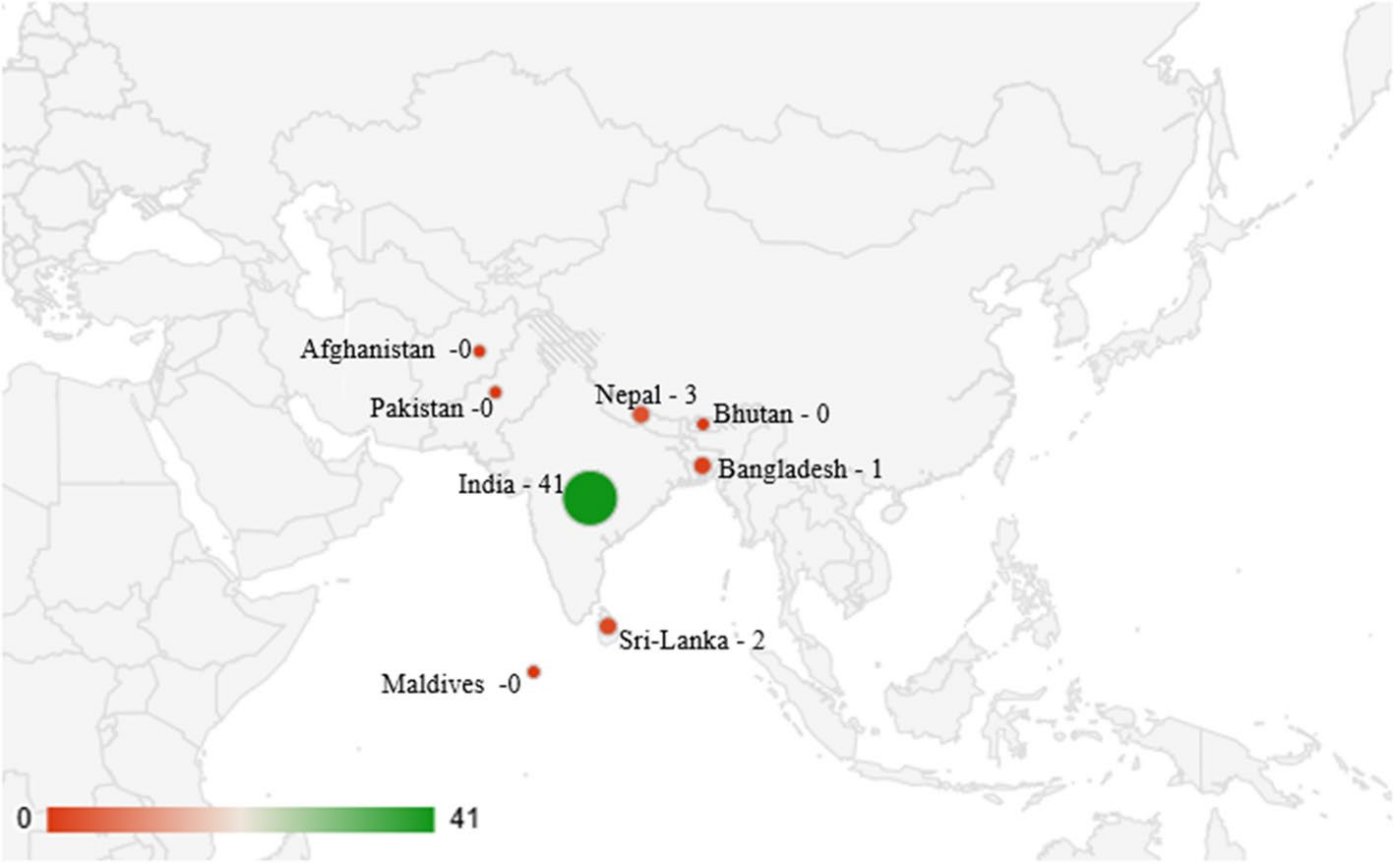
Geographical distribution of the studies among South Asian countries

The studies were conducted largely in hospital (*n* = 23), community (*n* = 18), school (*n* = 4), both hospital and community settings (*n* = 1) & both community and school settings (*n* = 1). Of the 47 studies, twenty-six were cross-sectional studies, seven were descriptive studies, five were qualitative studies, four were mixed method studies, three were case–control studies and one was a population study, and one used ex-post facto study design. Twenty-six studies were conducted in India; two studies were conducted in Sri Lanka and Nepal, respectively, and one study in Bangladesh. One of the studies was from a multi-country setting where India and Sri Lanka were included. Figure 2 shows the geographical distribution of the studies among South Asian countries. The population studied included spouses and adolescent children from families affected by individuals with problem drinking. There was a paucity of studies that focused on challenges faced by extended family members. A majority of the studies (*n* = 24) focused on women seeking treatment for their spouses diagnosed with alcohol dependency and the rest of the studies (*n* = 18) concentrated on spouses living with an alcoholic partner in the community. Five studies addressed school-going adolescent children affected by parental problem drinking. Table 2 (see Additional file 2) describes the characteristics of the included studies.

### Challenges faced by family members due to individuals with problem drinking

**Fig. 3:**
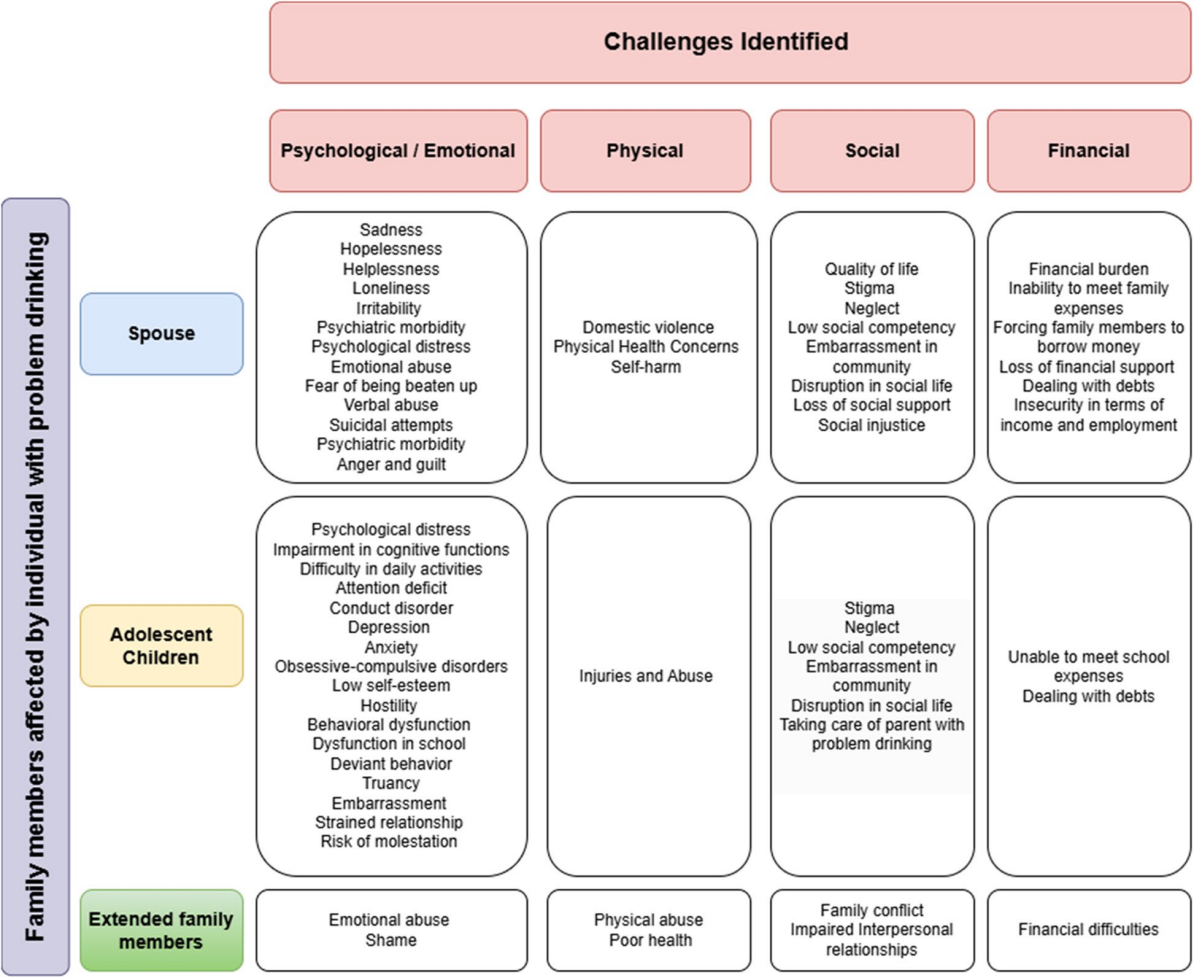
Challenges identified among the family members affected by individuals with problem drinking

Of the 47 studies, 37 studies described challenges faced by spouses while 10 studies described challenges among adolescent children in households with a person with problem drinking. Most of the studies identified physical and psychological/emotional challenges and fewer studies reported social and financial challenges. Figure 3 provides a visual representation of the challenges identified among the family members affected by individuals with problem drinking.

### Physical challenges

The review identified physical challenges faced by the spouses, adolescents, and extended family members of individuals with problem drinking. In the Indian context, studies conducted in Karnataka state reported physical issues among spouses and adolescents (ref of Karnataka articles). Two studies from Bengaluru, Karnataka specifically highlighted physical injuries and abuse experienced by adolescents, with considerable emotional distress [26, 35].

Wives reported physical problems, including abuse and domestic violence in the families due to husbands with problem drinking in studies done in Tamil Nadu [36–39]. Three studies out of six studies conducted in Maharashtra reported physical violence [19, 40, 41], whereas two studies also reported on sexual violence among wives of individuals with problem drinking [19, 40]. Spouses and adolescents reported physical and sexual abuse in the study conducted in Goa [42]. Problem drinking is linked to domestic violence, conflict within families, and social disruptions in a study done in West Bengal [43]. Prevalence of intimate partner violence [44], physical health concerns and harm towards spouses and their children were reported by studies done in Chandigarh [45]. Sleep disturbance among spouses [36, 45, 46] and adolescents [26, 47] was reported by two studies each respectively.

In a study conducted in Nepal, it was observed that women exposed to physical violence due to their partner's problem drinking were less likely to access and utilize skilled delivery assistance [48]. Another same study from Nepal identified spouses at the risk of being hypertensive due to a partner with problem drinking [49]. In Bangladesh, a study revealed physical challenges of spouses, such as domestic violence, non-consensual sex, and physical assault, mainly when husbands are intoxicated [50]. The study by Sorensen and colleagues emphasized the significant challenge of self-harm among women suffering from their partner's problem drinking in Sri Lanka [51].

### Psychological/emotional challenges

Spouses and children expressed their concern regarding psychological issues when dealing with individuals with problem drinking in a family context. Most studies included in the scoping review sourced from India, Nepal, Bangladesh, and Sri Lanka highlighted psychological challenges. Feelings of sadness, hopelessness, helplessness, loneliness, and irritability over minor things were expressed by spouses in a study done in Mangalore, Karnataka [52]. More than half of the spouses were diagnosed with psychiatric disorder/s and significant psychological distress, along with marital dissatisfaction, in a study conducted in Mysore, Karnataka [53]. Emotional violence was also reported in a community based study—conducted in Ramnagara district of Karnataka [54]. The six included studies addressing challenges of adolescents from Karnataka focused on psychological disorders, impairment in executive functioning, and difficulty in day-to-day functioning due to parental problem drinking. Problem drinking had a harmful impact on adolescents behavior, attention issues, conduct disorder, depression, anxiety, social phobia, obsessive compulsive problems, and low self-esteem, resulting in increased levels of psychological distress, somatization, and hostility [35, 47, 55].

Adolescent children living in families affected by parental problem drinking exhibited behavioural challenges that included trouble in school with teachers and peers which at times progressed to trouble with the law. The adolescents experienced difficulties related to selective attention, behaviour, emotional and cognitive regulation with deviant behaviour, conduct disorder, inattention, truancy, low self-esteem and low social competence being documented in children raised in such households [35, 47, 55–57]. Witnessing physical and verbal abuse from a parent with a problem drinking towards their mothers and siblings was distressing for the adolescents. They also suffered from embarrassment related to their fathers'drinking which affected routine school activities including peer relationships [26]. This caused them to drop out of school and resulted in low self-esteem and poor psychological well-being, occasionally leading to maladjustment. At times, the parent would demand that the adolescent buy alcohol from bars, causing them humiliation [26]. Despite these distressing living conditions and strained relationships, adolescents also expressed anxiousness regarding their parent with problem drinking which generally included the father [26, 58]. Gendered forms of risk were also captured in studies done in Karnataka in that female members of the family, particularly young girls were at risk of molestation and the drinking episodes spiraled into abusive situations including casting aspersions on their character [26, 47, 59].

Nine studies addressed spouses'psychological studies in the Tamil Nadu state of India. Spouses reported that fear of being beaten up or scolded caused them stress resulting in feelings of mental and emotional abuse. Misunderstandings and arguments, highlighting the crucial role of communication, ended in a breakdown in communication between family members. They also expressed that their children’s studies were affected as there was an emotional disturbance [37]. Most of the time, spouses were unaware of their husband’s drinking habits at the time of their wedding given the traditional practice of arranged marraige. Marital dissatisfaction was also expressed by spouses in the study conducted by Stanley S in Tamil Nadu [60]. Gupta and colleagues [61] stresses on the verbal abuse and suicidal attempts among spouses due to their partner’s problem drinking in Chennai, Tamil Nadu. Low level of perceived adjustment was observed in a community study concerning living with problem drinkers [58]. Spouses developed psychiatric disorders during their marital life. All the families had evidence of marital discord in the form of strained emotional bonding [62, 63]. Studies documented psychiatric morbidities among family members of problem drinkers, including social phobia, obsessive–compulsive problems, somatization, dysthymia, panic disorder, and single/multiple suicidal attempts, calling attention to the range of mental health concerns stemming from problem drinking, which were observed in Tamil Nadu [36, 39, 64]. In another community study conducted in Tamil Nadu, a decrease in quality of life was reported by wives of a persons with problem drinking [38]. Studies conducted in Maharashtra among wives of husbands with problem drinking also spoke about poor quality of life, humiliation or controlling behaviours from their husbands, abuse, forced to engage in sex against their will [40], hopelessness, threats and suicidal ideation [19, 65] and psychiatric morbidities among spouses [66].

Psychopathology was seen among adolescents of parents with problem drinking in studies done in Maharashtra [67] Goa [68] and Andhra Pradesh, India [69]. Common mental disorders, increased tension at home [70], and difficulty in relationships, shame, and abuse were also felt by affected family members [71] living with a person with problem drinking in studies conducted in Goa. In Ranchi, India, spouses of individuals with problem drinking reported significantly lower levels of expressiveness, achievement orientation, independence, and moral religious emphasis, resulting in disruptions in family cohesion, communication, and interpersonal communication in the family [72]. Studies from Kerala [73] and Punjab [74] reported domestic violence in most cases and at least one psychiatric morbidity including Major Depressive Disorder, anxiety disorders, and adjustment disorders. Two studies done in Chandigarh, India, wives of persons with problem drinking reported feeling afraid of their partners due to physical violence, threats, mental disturbance, and displacement of frustration on children, leading to neglect of children in the family [44, 45].

Chowdhury and colleagues [43] explained about wives bearing antisocial behaviour of individuals with problem drinking resulting in family discord in a study done in West Bengal, and disturbances in family functioning in a study done in Delhi [75].Another study conducted in India [76] emphasized on the caregiver burden experienced by spouses of individuals with problem drinking, impacting negatively on their marital relationship and wellbeing. One of the studies from Guwahati, India, reported that adolescents failed to develop an emotional bond with parents due to problem drinking in parent [77].

Callinan, S et al. [78], explained anxiety, depression, and reduced satisfaction with life among spouses due to their partners'harmful drinking in a multicounty study including Sri Lanka and India. In another study in Sri Lanka, it was noticed that the alcohol-related stressors exacerbated the non-alcohol-related daily life stressors within family relationships, resulting in conflict in partners and subsequent self-harm [51]. It was also noted that major depressive disorder in spouses of individuals with problem drinking is markedly higher in a study done in Sri Lanka [79]. Studies documented psychiatric morbidities among family members of problem drinkers calling attention to the range of mental health concerns stemming from problem drinking. In the study conducted in Nepal to study the association between intimate partner violence and alcohol use among partners in Nepal, it was found that women undergoing emotional and physical violence due to their partner’s alcohol use were less likely to attend the recommended antenatal visits [48]. Wives of husbands with problem drinking developed depressive disorder, conversion disorder, and anxiety disorder in a study done in Nepal [80]. The spouses were stressed by the ever-present threat of divorce and in several instances, polygyny was documented, which led to high levels of distress. These experiences cumulatively led to a communication breakdown. The study done in Bangladesh reported fear of the spouse/parent who was a problem drinker and the spouse who was the victim linked the fear to experiencing various forms of violence including child battery [50].

### Social challenges

The person with a problem drinking would be involved in accidents, at times passing out in public areas, and experience incarceration by law enforcement causing concern to family members [38, 45, 60] in studies done in Tamil Nadu, India. Failure to maintain social norms and practices by the problem drinker projected them as troublemakers engaging in deviant behaviors. Spouses experiencing the negative effects of problem drinking felt stigmatized and neglected [52] and felt low social competence [56]. This created instability and conflicts in society among community members in a study done in Bangladesh [50].

Spouses and adolescent children felt keen embarrassment in the community. The embarrassment spilled over into social functioning disrupting their routine social life. They felt shunned by society and relatives avoided them. Family members of the problem drinker also avoided religious events and social functions with consequent loss of social support [50]. The families collectively felt a sense of social injustice [51] and social liabilities were imposed on them leading to anger and guilt in a study conducted in Sri Lanka. It was concerning to note that some children and adolescents eventually progressed to imitate their parent's drinking behaviour [37].

### Financial challenges

Family members attributed problem drinkers'behavior to the immense financial burden on the family in included studies conducted in India, Sri Lanka, and Bangladesh. Problem drinkers would repeatedly borrow money and sell property to support their habit [37, 38, 60] reported in studies conducted in Tamil Nadu. The problem drinker frequently lost employment due to absenteeism leading to the economic crisis and inability to meet family expenses [51] in a study done in Sri Lanka. Spouses were deeply impacted by the crisis experienced by the family due to the pawning of valuables, destruction of household items, and forcing family members to borrow money for essentials, including buying alcohol in included studies conducted in Karnataka [26, 54], Delhi [75], West Bengal [43], Chandigarh [45] Goa [70]. There was also concern regarding the inability to repay money lenders which led to further refusal of availing loans. Problem drinkers were thought to use essential money meant for household use to support their habit and default on their payments. This dissuades moneylenders from lending money to anyone in an alcoholic household [37] in Tamil Nadu. They expressed concerns about not being able to pay school fees and meeting daily expenses at home. This compelled them to shoulder additional responsibility to contribute to the family income and sometimes have to take up multiple jobs. Women in their antenatal period who experienced violence had a lower likelihood of accessing delivery services [48] in a study done in Nepal. Wives of individuals with problem drinking in Bangladesh conveyed their economic crisis and expressed that they would never be in poverty if their husbands stopped spending on arrack drinking [50]. These aspects highlight the complex challenges that family members of problem drinkers face.

## Discussion

As per our knowledge, this scoping review is the first attempt to consolidate data available from family members affected by a person with problem drinking in the South Asian context. The available literature is predominantly from the Western context and largely leans towards the impact of parental alcohol use on adolescent drinking behavior; however, there is limited literature that maps the challenges faced by family members, including spouses, adolescent children, and extended family members, due to person with problem drinking in the South Asian context. Problem drinking causes harm to individuals closest to the problem drinker and has a wide range of consequences for families.

Family plays a pivotal role in the upbringing of adolescent children and needs to act as a buffer during challenging times [81]. As families of individuals with problem drinking face numerous challenges, it may be difficult for the adolescent living in the family to have normal functioning. They have risk for adverse outcomes due to parental drinking as observed by Rossow et al. [25]. While challenges faced by family members in the South Asian and western context were largely similar, in reviewing the articles several observations were made which also highlighted differences between the two contexts.

Problem drinking is often stigmatized and associated with shame, so families try to conceal it from others in the context of South Asia [82, 83]. In Western countries, alcohol consumption is normalized and accepted, though problem drinking may still have stigma. In the South Asian context, especially in traditional settings, alcohol consumption is less socially acceptable as compared to the West. In addition, religious and cultural practices also discourage, and, in some instances, they prohibit alcohol use [84]. Gender difference has been noted with respect to problem drinking in south Asia. The review brings to light that person with problem drinking in the south Asian countries were largely males i.e. fathers/husbands and it is rarely acknowledged among mothers/wives due to societal expectations [67].

In line with a previous review (Kaur & Ajinkya, 2014), we found that the families of persons with problem drinking are negatively impacted. Sarkar and colleagues described the challenges of spouses who constantly live in conflict. The family environment within a household affected by a person with problem drinking might be negative, hostile, critical, harsh, and rejectionist [27]. Through various studies exploring the psychological difficulties faced by spouses, we discovered that issues with problem drinking resulted in conflicts between the drinker and their spouses, as well as their adolescent children. This finding aligns with research conducted globally [85].

The maladaptive behavior of a problem-drinking partner affects spouses in a myriad of negative ways as it interrupts the normal functioning of the family. Our review found various psychiatric morbidity among spouses of persons with problem drinking. Most of the articles have focused on anxiety, depression, self-harm, and psychological problems like sleep deprivation and disturbances, diminished appetite, and weight loss, leading to physical maladies such as hypertension and somatization. Domestic violence on the part of the problem drinker towards the family members can take on many forms, including physical, emotional, or sexual abuse, which aligns with the current findings of the review. Eventually, intergenerational transmission of similar behavior may be handed down to the next generation, as observed in the literature [86].

In keeping with Murray Bowen’s theory, challenges reported in literature across South Asia were comparable; however, we also found instances of resilience among the spouses, including taking on additional responsibilities to tide over everyday challenges spawned by the partner’s problem drinking [37, 50]. It was also observed that parental responsibility was shouldered by adolescent children, depicting parentification owing to the crisis in the family [26]. Parentification refers to role reversal between the parent and child when it’s developmentally inappropriate, which includes management of household responsibilities with respect to handling life tasks or providing emotional support to another family member in the absence of the parent [15].

Child abuse, maltreatment, insufficient monitoring, and insufficient parental involvement were the challenges frequently observed in families contending with parental problem drinking in agreement with previous studies done in Finland [87, 88]. Moreover, adolescent children with parental drinking have lower levels of self-esteem and self-efficacy than adolescent children with non-drinking parents [23]. Significant behavioral differences are observed among adolescents with parents engaging in problem drinking. Male adolescents were prone to higher aggressive behavior while girls appeared to experience internalizing behaviors which is consistent with previous research done in high-income countries [89]. Irrespective of the culture and diverse population, problem drinking remains a challenge for the overall functioning of these families [90]. Compared to the west, research in this area is also limited in the eastern context. In Western countries, there is a greater emphasis on awareness and intervention for families affected with problem drinking. The limited studies that exist emphasize the [26]necessity for more specialized approaches to effectively address the needs of spouses and adolescents in South Asia. We found only two studies that focus on extended family members affected by individuals with problem drinking [37, 42]. This highlights the lack of research on how extended family members are also impacted.

### Strengths & limitations

A comprehensive search in six electronic databases helped to identify relevant articles to answer the research question. Two independent reviewers were engaged in the process of screening and conflict resolution. Although we extensively searched various databases, this review has some limitations. Our search included studies across south Asian countries; however, we found that most of the studies were from the Indian context. We included only English language literature, which could have resulted in missing out on studies published in the regional language.

## Conclusion

This scoping review examined the various challenges and consequences faced by families affected by individuals with problem drinking. It highlighted the complexities associated with problem drinking, which can lead to strained family dynamics, inadequate parenting, emotional distress, and mental health issues. The psychological, emotional, and social burdens experienced by adolescent children and spouses can significantly affect their overall well-being throughout their lives. Healthcare professionals should consider the challenges impacting the entire family while also addressing the needs of each individual involved in managing someone with problem drinking.

## Supplementary Information

Supplementary Material 1.

Supplementary Material 2.

Supplementary Material 3.

## Data Availability

All data generated for the review are available upon request from the authors.
