# Supplementary material for: Mapping the journey of families navigating problem drinking in South Asia: a scoping review
Source: BMC Public Health. 2025 May 9;25:1715. doi: 10.1186/s12889-025-22967-y (PMC12063230; doi:10.1186/s12889-025-22967-y)
Supplement: Supplementary file 1 — Supplementary Material 1. [file 12889_2025_22967_MOESM1_ESM.docx]

| Date of search | Search engine /Database | Search strategy | Number of hits |
| --- | --- | --- | --- |
| 27-06-2023 | PubMed (Medline) | (("challenge"[All Fields] OR "challenges"[All Fields] OR ("difficulties"[All Fields] OR "difficulty"[All Fields]) OR ("issue"[All Fields] OR "issues"[All Fields]) OR ("effect"[All Fields] OR "effects"[All Fields]) OR ("negative outcomes"[All Fields] OR ("influence"[All Fields] OR "harmful outcome"[All Fields] OR ("impact"[All Fields] OR "consequence*"[All Fields] OR ("risk factors"[MeSH Terms] OR "risk factors"[All Fields]) OR "affect*"[All Fields] OR ("adverse effects"[MeSH Subheading] OR "adverse effects"[All Fields]) OR ("spouse abuse"[MeSH Terms] OR "spouse abuse"[All Fields] OR "partner abuse"[All Fields] OR "spousal abuse"[All Fields]) OR "wife abuse"[All Fields]) OR "substance related disorders"[MeSH Terms] OR "substance related disorders"[All Fields] OR "abuse"[All Fields]) OR ("domestic violence"[MeSH Terms] OR "domestic violence"[All Fields]) OR ("intimate partner violence"[MeSH Terms] OR "intimate partner violence"[All Fields]) OR "intimate partner abuse"[All Fields] OR "injuries"[MeSH Subheading] OR "injuries"[All Fields] OR "wounds and injuries"[MeSH Terms] OR "wounds and injuries"[All Fields] OR ("gender based violence"[MeSH Terms] OR "gender based violence"[All Fields] OR ("family conflict"[MeSH Terms] OR "family conflict"[All Fields] OR "marital discord"[All Fields]) OR "marriage"[MeSH Terms] OR "marriage"[All Fields] OR "interparental conflict"[All Fields]) OR ("sex offenses"[MeSH Terms] OR "sex offenses"[All Fields] OR "sexual violence"[All Fields]) OR "sexual abuse"[All Fields]) OR ("rape"[MeSH Terms] OR "rape"[All Fields]) OR ("assault"[All Fields] OR "assaulted"[All Fields] OR "assaults"[All Fields]) OR "employment"[MeSH Terms] OR "employment"[All Fields] OR "income"[All Fields] OR "wages"[All Fields] OR "financial instability"[All Fields] OR "poverty"[All Fields] OR "economic hardship"[All Fields] OR "financial stress"[MeSH Terms] OR "financial stress"[All Fields] OR "financial burden"[All Fields] OR "economic burden"[All Fields] OR "financial challenges"[All Fields] OR "separated"[All Fields] OR "separation"[All Fields] OR ("interpersonal conflict"[All Fields] OR "conflict, psychological"[MeSH Terms] OR "psychological conflict"[All Fields] OR "conflict"[All Fields] OR ("childhood abuse"[All Fields] OR "substance related disorders"[MeSH Terms] OR "substance related disorders"[All Fields] OR ("child abuse"[MeSH Terms] OR "child abuse"[All Fields] OR "child mistreatment"[All Fields]) OR "child maltreatment"[All Fields] OR "child neglect"[All Fields]) OR ("child abuse, sexual"[MeSH Terms] OR "sexual child abuse"[All Fields] OR "child molestation"[All Fields]) OR ("aggression"[MeSH Terms] OR "aggression"[All Fields] OR "aggressive behavior"[All Fields]) OR "aggressiveness"[All Fields] OR ("antisocial personality disorder"[MeSH Terms] OR "antisocial personality disorder"[All Fields] OR "antisocial behavior"[All Fields]) OR ("conduct disorder"[MeSH Terms] OR "conduct disorder"[All Fields]) OR ("attention deficit disorder with hyperactivity"[MeSH Terms] OR "attention deficit disorder with hyperactivity"[All Fields] OR "attention deficit hyperactivity disorder"[All Fields]) OR "emotions"[MeSH Terms] OR "emotions"[All Fields] OR "emotion"[All Fields] OR ("social behaviour"[All Fields] OR "social behavior"[MeSH Terms] OR "social behavior"[All Fields]) OR ("mental health"[MeSH Terms] OR "mental health"[All Fields]) OR "emotional disturbances"[All Fields] OR ("physical abuse"[MeSH Terms] OR "physical abuse"[All Fields]) OR ("traumatic"[All Fields] OR "traumatized"[All Fields] OR "traumatizing event"[All Fields] OR ("traumatizing experience"[All Fields] OR ("stress"[All Fields] OR "stressful"[All Fields] OR ("stress disorders, traumatic"[MeSH Terms] OR "traumatic stress disorders"[All Fields] OR ("mental disorders"[MeSH Terms] OR "mental disorders"[All Fields] OR "mental illness"[All Fields]) OR ("depressed"[All Fields] OR "depression"[MeSH Terms] OR "depression"[All Fields] OR "depressive disorder"[All Fields] OR ("psychological distress"[MeSH Terms] OR "psychological distress"[All Fields] OR "emotional distress"[All Fields]) OR ("stress, psychological"[MeSH Terms] OR "psychological stress"[All Fields] OR "depressive symptoms"[All Fields]) OR "emotional depression"[All Fields]) OR ("behavior"[MeSH Terms] OR "behavior"[All Fields] OR "behavioral"[All Fields] OR "behavioural"[All Fields] OR ("problem behavior"[MeSH Terms] OR "problem behavior"[All Fields] OR "behavioural problems"[All Fields]) OR ("anxiety"[MeSH Terms] OR "anxiety"[All Fields] OR "social anxiety"[All Fields]) OR "nervousness"[All Fields] OR "anxiousness"[All Fields]) OR ("personality disorders"[MeSH Terms] OR "personality disorders"[All Fields] OR "personality disorder"[All Fields]) OR "avoidant personality disorder"[All Fields]) OR ("quality of life"[MeSH Terms] OR "quality of life"[All Fields]) OR "low self-esteem"[All Fields])) OR ("delinquencies"[All Fields] OR "delinquency"[All Fields] OR "delinquent"[All Fields] OR ("substance related disorders"[MeSH Terms] OR "substance related disorders"[All Fields] OR ("underage drinking"[MeSH Terms] OR "underage drinking"[All Fields] OR "adolescent drinking"[All Fields]) OR "academic performance"[All Fields]) OR "disruptive behaviour"[All Fields] OR ("self efficacy"[MeSH Terms] OR "self efficacy"[All Fields]))) OR "family violence"[All Fields]) OR ("suicidal ideation"[MeSH Terms] OR "suicidal ideation"[All Fields])) AND ("spouses"[MeSH Terms] OR "spouses"[All Fields] OR "spouse"[All Fields] OR "married persons"[All Fields] OR "married person"[All Fields] OR "husbands"[All Fields] OR "husband"[All Fields] OR "domestic partners"[All Fields] OR "domestic partner"[All Fields] OR "wife"[All Fields] OR "wives"[All Fields] OR "Adolescent"[MeSH Terms] OR "Young Adult"[MeSH Terms] OR "Family"[MeSH Terms] OR "Family"[All Fields] OR "familial"[All Fields] OR "families"[All Fields] OR "family members"[All Fields] OR "family member"[All Fields] OR "relative"[All Fields] OR "relatives"[All Fields] OR "family research"[All Fields] OR "Extended Family"[MeSH Terms] OR "Extended Family"[All Fields] OR "extended families"[All Fields] OR "family extended"[All Fields] OR "multigenerational family"[All Fields] OR "family multigenerational"[All Fields] OR "household"[All Fields] OR "extended household"[All Fields] OR "multigenerational household"[All Fields] OR "multigenerational households"[All Fields]) AND ("Alcohol Dependence"[All Fields] OR "Alcohol Addiction"[All Fields] OR "Chronic Alcoholic Intoxication"[All Fields] OR "Alcohol Abuse"[All Fields] OR "Alcohol Use Disorder"[All Fields] OR "Alcohol Use Disorders"[All Fields] OR "harmful drinking"[All Fields] OR "hazardous drinking"[All Fields] OR "alcohol dependence disorder"[All Fields] OR "Alcohol Related Disorders"[All Fields] OR "Alcohol Problem"[All Fields] OR ("alcoholic intoxication"[MeSH Terms] OR "alcoholic intoxication"[All Fields] OR "drunkenness"[All Fields] OR "drunken"[All Fields]) OR "Alcohol drinking"[All Fields] OR "paternal alcohol use"[All Fields] OR "maternal alcohol use"[All Fields] OR "fathers drinking"[All Fields] OR "mothers drinking"[All Fields] OR "Paternal drinking"[All Fields] OR "maternal drinking"[All Fields] OR "alcoholics"[MeSH Terms] OR "alcoholics"[All Fields] OR "alcoholic"[All Fields] OR "alcoholism"[MeSH Terms] OR "alcoholism"[All Fields] OR "Alcohol Intoxication"[All Fields] OR "parental alcoholism"[All Fields] OR "harmful alcohol use"[All Fields] OR "alcohol consumption"[All Fields] OR "parental alcohol use"[All Fields] OR "parental binge drinking"[All Fields] OR "alcoholic parents"[All Fields] OR "Parental problem drinking"[All Fields] OR "heavy drinking"[All Fields] OR "parental alcohol use"[All Fields] OR "drinking parents"[All Fields] OR "problem drinkers"[All Fields] OR "problem-drinking mother"[All Fields] OR "problem-drinking father"[All Fields] OR "Parental drunkenness"[All Fields] OR "Parental alcohol misuse"[All Fields] OR "parental intoxication"[All Fields] OR "parental alcoholism"[All Fields] OR "fathers alcohol problems"[All Fields] OR "problematic drinking"[All Fields]) AND ("South Asia"[All Fields] OR "Southern Asia"[All Fields] OR "Asia south"[All Fields] OR "British Indian Ocean Territory"[All Fields] OR "Afghanistan"[MeSH Terms] OR "Afghanistan"[All Fields] OR "Pakistan"[MeSH Terms] OR "Pakistan"[All Fields] OR "Bangladesh"[MeSH Terms] OR "Bangladesh"[All Fields] OR "Bhutan"[MeSH Terms] OR "Bhutan"[All Fields] OR "Sri Lanka"[MeSH Terms] OR "Sri Lanka"[All Fields] OR "Nepal"[MeSH Terms] OR "Nepal"[All Fields] OR "Maldives"[MeSH Terms] OR "Maldives"[All Fields] OR "india"[MeSH Terms] OR "india"[All Fields] OR "asia, southern"[MeSH Terms])) AND (english[Filter]) | 1750 |
|  |  |  |  |
