# Supplementary material for: Mapping the journey of families navigating problem drinking in South Asia: a scoping review
Source: BMC Public Health. 2025 May 9;25:1715. doi: 10.1186/s12889-025-22967-y (PMC12063230; doi:10.1186/s12889-025-22967-y)
Supplement: Supplementary file 2 — Supplementary Material 2. [file 12889_2025_22967_MOESM2_ESM.docx]

**Table 2: Characteristics of included studies**

|  | | | | **Population** | | | **Challenges identified** | | | |
| --- | --- | --- | --- | --- | --- | --- | --- | --- | --- | --- |
| [S.No.](http://s.no/) | Author ID | Study setting | Study design | Participant description | Sample Size | Place (Country) | Physical | Psychological/Emotional | Social | Financial |
| 1 | Shiji PJ et al., 2017 | Deaddiction centre, private hospital | Descriptive survey/ Descriptive correlational research design | Spouse (wives of alcoholics whose husbands are admitted to the selected de-addiction centre for treatment of alcoholism) | 30 | Mangalore, Karnataka, India | • Disturbed sleep | • Feelings of sadness, loneliness, helplessness, and hopelessness • Getting irritated over silly things | Neglected in the community | NA |
| 2 | Olickal J et al., 2022 | urban and rural field practice areas of a tertiary care centre | Concurrent mixed method study [cross sectional study & 4 FGDs] | Men aged above 18 years in quantitative study, Alcohol  dependents and their family members participated in the qualitative study | 316 for cross sectional, 14 alcohol users and 16 family members participated in the  FGDs, in which 8 alcohol users and 10 family members were from  the rural areas. | Puducherry,Tamil Nadu, India | Physical abuse | Mental, emotional and stress. Fear of getting beaten-up or scolded and the development of arguments and fights with them also gave them additional stress and burden. Children's studies are affected as they got emotionally disturbed. Child imitating father's behaviour of consuming alcohol at home reported by a middle-aged woman. A few female participants stated that the alcohol users gave them unnecessary hindrance, annoyance and tortured them with abusive language whenever they came home under its influence. Misunderstandings and arguments popped up frequently and led to breakdown in communication between them and strained family relationships. This study found that there was quarrelling with wives and children after the intoxication of alcohol. | NA | One of the participants stated, “Because of my husband’s habit, some of the money lenders refuse to lend me money, quoting that my husband will waste all the money to drink and will not repay it correctly on time. Hence, I could not even borrow money when in need. |
| 3 | Omkarappa DB et al., 2019 | Government high school | Cross‑sectional comparative study | 100 children from alcoholic parents and 100 children from nonalcoholic parents | 200 | Bengaluru, Karnataka, India | Physical injury | Anxiety, depression, self-esteem, separation anxiety, social phobia, obsessive compulsive problems | NA | NA |
| 4 | Akombi-Inyang, B.,et al.,2021 | 2011 and 2016 Nepal Demographic and Health Surveys (NDHS) | Cross sectional study | A total of 3067 women who interviewed for domestic violence module and had most recent live birth 5 years prior surveys were included in the analysis. | 25536 women (12,674 women in 2011 NDHS and 12,862 in 2016 NDHS) | Nepal | Physical violence, sexual violence. Women exposed to physical violence were less likely to access and utilize skilled delivery assistants. | Emotional violence. Women who reported experiencing emotional or physical violence with a partner who consume alcohol were less likely to attend the recommended antenatal visits. | NA | NA |
| 5 | Nattala, P et al., 2022 | School and community | Qualitative study design(IDI) | 39 AOFADs and 45 adolescent offspring of fathers without a history of alcohol-use disorders | 84 | Bangalore, Karnataka, India | NA | delinquent behavior, attention difficulties, and conduct disorder, depression, anxiety, and low self-esteem, heightened levels of psychological distress, somatization, hostility, and anxiety | impact on the overall well-being and adjustment | NA |
| 6 | Dayananda BO et al., 2018 | Three government high schools | Cross-sectional descriptive design | Children of alcoholic parents | 100 | Bangalore, Karnataka, India | Problems like physical symptoms and sleep disturbance. | mild to moderate impairment in psychosocial functioning. Significant impairments with anxiety and/or depression, significant problems with conduct, significant impairments in attention. | NA | NA |
| 7 | Shekhawat BS et al., 2017 | Tertiry care hospital | Cross‑sectional study | 60 alcohol dependent husbands & their wives | 120 (60 alcohol dependent & 60 heroin dependent patients & their wives) | India | NA | Caregiver burden [impact on wellbeing, impact on marital relationship, appreciation of caregiving, impact on relation with others, perceived severity of disease] | NA | NA |
| 8 | Indu PV et al., 2018 | Deaddiction centre of a Tertiry care hospital | Cross‑sectional study | Spouses(wives) | 60 | Kerala, India | NA | Domestic violence was reported by 41 out of 60 cases. At least one psychiatric morbidity was observed in 51 cases–MDD in 15, anxiety disorders in 6, and adjustment disorder in 32 cases each. | NA | NA |
| 9 | Nattala, P et al., 2022 | outpatient services and snow bowling | Qualitative study design(IDI) | Adolescent children | 15 | Bangalore, Karnataka, India | •Experiences physical abuse •Being turned out of the house •Physical abuse with considerable emotional distress •Disturbances in appetite, sleep •Feeling tired and exhausted | • Witnessing physical & verbal abuse towards mother & siblings • Risk of being molested when alone at home, particularly girls • Boys spoke about him in angry, grim, clipped tones • Verbal abuse,  • A particular form of verbal abuse reported by five girls involved casting aspersions on their character • Embarrassment and distress related to their fathers’ drinking • Feelings of anxiety and worry about father’s health apparently troubled some respondents, About half also expressed worry and anxiety about the well-being of other family members, especially their mother Fear of parents separation · Risk of being molested when alone at home, particularly girls | being forced to go to the shop to buy alcohol for their father and get humiliated at the shop by other men, to help the family, because father was irresponsible, they avoided people, family and social functions, places of worship, dropped out of school/college, as father was not supporting the family Social impact(embarrassment, distressed by others reactions by their fathers, relatives not visiting them anymore, social liability imposed by them) | Witnessing destructive behavior(destroying things at home, causing financial loss),Narrative accounts included dealing with debts created by their father, father using up money required for family expenses, including pawning or selling of household articles and kitchen provisions, and not paying school fees, to shoulder responsibilities, having to look after the father when he is not well due to drinking, going out in search of him or dragging him back to the house after finding him lying unconscious on the streets, |
| 10 | Hazarika M et al., 2018 | Treatment centre and community | Case-control study | Four groups of 50 subjects in each group comprising alcohol dependent fathers (ADF), non-alcohol dependent fathers (NADF), sons of ADF (SADF), and sons of NADF (SNADF). The ADF were from treatment centres located in Guwahati, Assam and the NADF were from the community | 200 | Guwahati, Assam, India | NA | when alcoholism was present, the children in a very consistent manner failed to develop emotional ties with either parent. | NA | NA |
| 11 | Callinan GS et al., 2018 | GENAHTO Project (community) | Cross‑sectional study | 10,613 female respondents, 7,091 with intimate live-in partners - Sri Lanka: 1261, India: 1780 | Sri Lanka: 1261, India: 1780 | 9 countries {Australia, United States, Ireland, Thailand, Sri Lanka, India, Nigeria, Vietnam, Lao PDR} | NA | Respondents with a harmful partner were significantly more likely to experience anxiety and depression and report reduced satisfaction with life in Sri Lanka and India. Interestingly, it is worth noting that the reported incidence of anxiety or depression or satisfaction with life in those with no partner was often similar to those with a harmful drinking partner, indicating that having a harmful heavy drinking partner negated the benefits of having a partner in the first place. | NA | NA |
| 12 | Wagman AJ., 2018 | Three large UHCs in three major slum communities | Cross‑sectional study | Women (postpartum women seeking immunization for their infant ≤6 months of age) | 1038(husband's use of alcohol & postpartum IPV:sub-sample 936 women) 95 provided consent & completed the survey | Mumbai, India | Postpartum IPV and maltreatment | NA | NA | NA |
| 13 | Saxena S et al., 2003 | Poor urban community (slum) | Qualitative study (IDI) | Wives of men using alcohol (Families with at least one adult consuming alcoholic drink at least 3 times every week in the previous 1 month were included in group A. Families with no adults consuming alcoholic drinks more than once in the last one month were included in group B) | Group A(wives of men with alcoholics): 98, Group B: 99 | Delhi, India | The more heavily drinking group A was more likely to report major illnesses or injuries during the past 1 year and was more likely to require medical treatment | Significant differences in two groups: on the health of the user; family relationships; children’s upbringing; and social relationships. | NA | Group A, on an average, spent almost 14 times more on alcohol per month compared with group. A larger proportion of families in group A (54 vs 29) had significant debt compared with group B. |
| 14 | Stanley S et al., 2012 | Deaddiction facility non-government | Cross sectional study | 150 wives of alcoholics with an equal number of wives of alcoholics | 150 wives of alcoholics | Tiruchirappalli, Tamil Nadu, India | Beating wife and children | Wives were unaware of their husband's drinking habits during their marriage. Quarrelling with relatives and arguments with neighbours. Verbal abuse. All of the marital adjustment subdimensions (consensus, cohesiveness, affectional expression, and marital satisfaction) were significantly lower for wives of persons with problem drinking than for the reference group. Wives of person with problem drinking show worse reinforcement, role, communication, cohesiveness, social support, and leadership scores (FIPS subdimensions) than the reference group. | Other reported behaviors included being involved in accidents, getting into trouble with the police, blacking out in public areas, and getting into accidents. | The husband's drinking was blamed for their family's financial problems. Wives said that their spouses (person with problem drinking) frequently borrowed money and sold property to fund their drinking habits. |
| 15 | Gupta A et al., 2015 | Community based | Cross sectional study | Men & women in the same household | 1053 | Chennai, Tamil Nadu, India | NA | Verbal abuse, suicidal attempts | NA | NA |
| 16 | Sharma,N et al., 2016 | Deaddiction centre | Descriptive study | Spouse (wives) | 30 | Chandigarh, India | • Ignoring physical health • Sleep disturbance • Physical violence • Physical harm towards children | • Mental disturbance • Feelings of anxiousness and frustration • Displacement of frustration on children and ignoring children | • Decline in social relationships • Reduced social visits • Feeing ashamed in society | • Only 3 spouses out of 30 report financial problems |
| 17 | Karim, K. R. (2006) | Community | Quantitative and qualitative (interview) | Married male arrack drinkers as well as their wives | 100 | Parbattipur, Bangladesh | frequently beaten by their husbands, Striking women by hands, pitching by catching hair, spitting on women’s body, kicking on women’s body, beating by stick or rods, forced into unwilling sex, biting/hitting sex organs, forced into deviant sex | Violently assaulted, behavioral abnormalities in husbands, Due to their behavioral abnormalities, arrack drinkers are not always able to carry out familial responsibilities properly. They are also irresponsible in their own occupations. Many of them often remain absent from work while they feel sleepy and tired because of drinking. Men drinkers spend a good amount of their family income for arrack drinking purposes. They seldom care for household economy. Verbal rebuke uttering profanities, profanities/insults on wives' parents, demanding dowry and ornaments, threat of divorce/polygyny marriage, Destruction of properties, domestic animal battering, domestic worker battering, abuse of children | Arrack drinkers also create social problems through their different deviant behaviors as they often fail to maintain standard social norms and practices. They often get involved in unethical and anti-social activities that create social tensions and unrest among other social members. They create problems in attending social functions and ceremonies while most of them (arrack drinkers) are not able to maintain their normal patterns of behaviors | In contrast, 97% of the wives reported that they face economic crisis due to their husbands’ drinking spending. Many wives also mentioned that they would never be in poverty if their husbands could stop spending money on arrack drinking |
| 18 | Nayak, MB et al., 2010 | Rural and urban areas | Population study | women who provided adequate information on both their own and their partner’s alcohol use | 821 | Goa, India | NA | common mental health disorders among adult women. Increased tension at home, threat to partner's health, partner alcohol use-related accidents and legal problems, partner drinking-related fights or that they or their children were physically hurt by a drinking partner | NA | financial problems |
| 19 | Bhattacharjee D et al., 2013 | Hospital | Case control study | 30 spouses of patients diagnosed with alcohol dependence syndrome (ICD-10 DCR) and 30 spouses of normal controls (GHQ-12 score <3) | 60 | Ranchi, Jharkhand, India | NA | Lower on cohesion, expressiveness, independence, achievement orientation, intellectual cultural orientation, active recreational orientation, moral religious emphasis and organization. | NA | NA |
| 20 | Thomas DS et al., 2012 | Hospital | Cross-sectional design | 60 children (10 to 14 years) of alcoholic fathers | 60 | Mangalore, Karnataka, India | Physical problems (62.6%) | Psychosocial problems (60.829%) and school problems (51.472%) | NA | NA |
| 21 | Kishor, M et al., 2013 | Hospital | Cross-sectional design | Men with Alcohol Dependence Syndrome (ADS) and their Spouses (wives) | 60 | Mysore, Karnataka, India | NA | More than half of the spouses (65%) had a psychiatric disorder. Primarily mood and anxiety disorder were present. Major depressive disorder was present in 43%. Psychiatric morbidity, marital dissatisfaction in spouses and higher adverse consequences alcohol dependence in their husbands, were found to be significantly correlated with each other and their association was strong. | NA | NA |
| 22 | Singh, R et al., 2022 | Psychiatry department of tertiary care centre | Cross-sectional design | Alcohol Dependent patients and their family members including spouses and children | 167 [patients diagnosed with Alcohol dependence(50) & **their spouses(50)** & children(67) | Punjab, India | NA | spouses - psychiatric morbidity (68%), the commonest was major depressive episodes ; Generalized anxiety disorder(22%), Dysthymia(6%), Panic disorder(6%) | NA | NA |
| 23 | Dandu A et al., 2017 | Hospital | Cross‑sectional study | Spouses(wives) | 101 | Tirupati, Andhra Pradesh,  India | NA | The psychiatric morbidity was more in 1–10 years duration of marital life (30.7%). psychiatric morbidity was more among women who received the verbal and physical violence constituting for about 37.6%. Among the study subjects, 44.6% had depressive disorders, 3% had anxiety disorders, 18.8% had adjustment disorders, and 33.7% had no psychiatric disorders. | NA | NA |
| 24 | Patkar, P et al., 2021 | Hopital | Cross‑sectional study | Wives of men with alcohol dependence syndrome (diagnosed as per ICD 10) & age‑matched control group of 50 wives of nonalcoholic men without any medical comorbidities. | 100 | Maharashtra, India | NA | poor quality of life, higher levels of depression, suicidal ideation | NA | NA |
| 25 | Omkarappa DB et al., 2019 | Government high school | Cross‑sectional study | 100 children of alcoholics | 100 | Bengaluru, Karnataka, India | NA | Social competence | NA | NA |
| 26 | Datta BK et al., 2020 | Data from 2016 Nepal Demographic and Health Survey (2016 DHS) | Cross‑sectional study | women whose husbands drink alcohol and in those whose husbands do not | 3778 | Nepal | Higher risk of being hypertensive | NA | NA | NA |
| 27 | Nadkarni A et al., 2017 | Community gate keepers | Mixed methods study with multiple steps: qualitative in-depth interviews (IDI) in step one were followed by an intervention cohort with before and after design in step two. | Affected family members | 30 | Goa, India | Physical abuse, sexual violence | NA | NA | NA |
| 28 | Berg MJ et al., 2010 | Low income community | Mixed method study [married men - survey data; married women - observational & interview data (In depth ethnographic interviews)] | Married men living with their wives, married women (married on average 14.4 years) | Married men - 486(survey), **married women - 44(IDIs)** | Navi Mumbai, Maharashtra, India | Physical violence. physical violence was also reported to occur when families experienced financial pressure, the wife refused to have sex, and when the husband blamed his wife for the children’s misbehaviors or illnesses. Sexual violence | Verbal (89% of all cases); but verbal violence often co-occurs with physical violence (82% of all cases), psychological violence includes instances of husbands humiliating or controlling their wives - "He did not allow me to go anywhere. He never takes me for outing or anything. Whenever he has a holiday, he goes alone but never takes me with him. In the beginning I asked him but after that I never talk about this. When I asked him, he shouted at me and abused me. So, after that I never asked him". Women often reported being forced to engage in sex against their will. | NA | NA |
| 29 | Kadam K S et al., 2021 | Hospital | Cross sectional study | Spouses(wives) | 100 | Mumbai, Maharashtra, India | physical violence, sexual aggression | Perceived stress, domestic violence, suicidal ideation & behaviour, threats | NA | NA |
| 30 | Sorensen JB et al., 2017 | Community | Qualitative study design (IDI & FGD) (Narrative life story interviews) and FGD | Narrative life story interviews - 19 individuals who had self-harmed where alcohol was involved and 25 of their relatives: 10 FGDs - gender & age segregated groups | 44 + (10 FGD) | Sri Lanka |  | Admitted to hospital having self-harmed by swallowing animal medication, her third incidence of self-harm happened after a violent conflict between the spouses over a broken phone. Everyday life appeared stressful, strained financially, her family had not been able to complete building their house. Husbands drinking often led to domestic violence and financial difficulties, and alcohol-related, abuse by Husband, submissive role by wives, stressor, women ingesting pesticides. self-sacrifice | injustice, need to remain silent when spouses drink | insecurity in terms of income and employment. |
| 31 | Ariyasinghe et al., 2015 | Community setting | Cross sectional study | Of those 202 men who used alcohol, 168 were married to or cohabiting with women. For the present study, researcher visited each of these 168 households to recruit the eligible women. | 156 | Sri Lanka | NA | The prevalence of MDD among spouses of men who use alcohol is markedly higher | NA | NA |
| 32 | Bagul et al., 2015 | Psychiatric dept, Hospital setting | Cross Sectional Observational Study | 60 spouses of males with ADS | 60 | Maharashtra, India | NA | Dysthymic disorder, Major Depressive Disorder, Panic Disorder, Generalized Anxiety Disorder, Specific Phobia, Adjustment Disorder with depressed mood, with mixed anxiety and depressed mood. Adverse consequences of alcohol and marital quality with psychiatric morbidity in spouses. | NA | NA |
| 33 | Behera et al., 2022 | School setting | Descriptive Survey cum Correlational design method. | 30 from alcoholic and 30 non-alcoholic groups in each rural and urban locality | 120(from 6 schools) | Orissa, India | NA | NA | Low social competence | NA |
| 34 | Chowdhury et al., 2006 | Community setting | Qualitative method (88 IDIs & 10 per village (6 villages) FGD) | People from community belonging to 6 villages (Sundarban Delta, West Bengal) | Not reported | The Sundarban, West Bengal, India | Physical‘ ‘torture’’ He is despicable and I wish that he would die! He has made my life miserable and even now *he beats me up.* He returns home drunk and creates a lot of commotion at night. Our children are grown up, and even though they are aware of what is happening, this doesn’t stop him. I wish he would never return home. It hardly matters whether he lives or dies. He collects tiger prawn seedling, and he spends all his earnings on drinks. He doesn’t contribute anything towards running the household. | Disrupting family life. Marital conflict and conflicts with in-laws. Family conflicts, and criminality. *A chronic drunkard of "Phulbari pawns the cooking vessels when he needs money to drink". When his wife protested, he beat her.* A year earlier, in an inebriated state, he set fire to the thatch of his hut. Some women also feared health effects, that their husbands might die from drinking. Women typically bear the brunt of antisocial behaviour from problem drinking. Mental torture. Promiscuity, extramarital relations, and wife beating are the direct consequences of alcohol abuse. | NA | Drinking cost affecting  domestic finances. Economic distress in the family. |
| 35 | Deepika KS et al, 2017 | Tertiary care Psychiatric Hospital | Prospective case control study | Adolescent children of men with alcohol dependence syndrome (ICD 10) | Adolescents 13-18 years, Study group had 24 and control group had 25 | Goa, India | NA | Higher prevalence of psychopathology in study group | NA | NA |
| 36 | Govindappa, L et al., 2014 | Community setting | Descriptive study | Wives of alcoholics living in 15 villages - 58 households had person with alcohol use - 50 households gave consent | 50 | Ramnagara, Karnataka, India | Physical violence | A community study on violence among wives of alcoholics | NA | Economic violence |
| 37 | Jenefer, M., 2016 | Community setting | Descriptive study | - | 30 from 13 – 14 years and 35 from  15 – 16 years and 17 – 18 years respectively | Coimbatore, Tamil Nadu, India | NA | Low level of perceived adjustment | NA | NA |
| 38 | Lingeswaran A et al., 2016 | Hospital setting | Cross sectional study | Sixty‑two alcohol‑dependent males, their spouses (62) and children (110). |  | Puducherry,Tamil Nadu, India | NA | Wives of alcohol dependent husbands  Bipolar affective disorder  Major depressive disorder  Dysthymia  Anxiety disorders Somatoform disorder  Disorders of sexual dysfunction Emotionally unstable personality traits  Histrionic personality traits  Marital disharmony in the form of strained emotional and physical bonding. Extramarital relationships | NA | NA |
| 39 | Mammen et al., 2015 | Hospital setting | Observational and cross sectional study | spouses of 100 male patients with a diagnosis of alcohol dependence syndrome according to DSM IV TR criteria | 100 | Pondicherry, Tamil Nadu, India | NA | Prevalence of mood disorders, Mixed Anxiety Depression, Adjustment Disorder, Somatization Disorder, Panic Disorder, Generalized Anxiety Disorder | NA | NA |
| 40 | Manohar et al., 2010 | Hospital setting | Cross section survey (retrospective design) | Alcoholic patents visiting to the department of psychiatry with their wives for getting treated for alcohol dependence syndrome and the wives of other psychiatric patients visiting to the department of psychiatry with their wives were taken as sample for the study32 wives of alcoholic patients and 32 non-alcoholics patients | 64 | Salem, Tamil Nadu, India | Domestic violence | Unhappy feelings, frequent suicidal thoughts, strong desire to die, planned a time for committing suicide, self-harming & changed things due to the self-harm, anything stopped from killing yourself, and what they could look forwarded to the future respectively. The wives of alcoholics might have more depressive features than the wives of non-alcoholics. | NA | NA |
| 41 | Parthasarathy K et al., 2013 | Community setting | Descriptive study | 22 respondents (wives of alcoholics) from each slum areas (n=110) | 110 | Tiruchirappalli, Tamil Nadu, India | Physical problems | Affective problems, Ego problems, low level of quality of life | Social dysfunction | Economic problems |
| 42 | Sedain et al., 2013 | Psychiatric department of CMC Teaching Hospital, Bharatpur, Nepal | Cross sectional study | Spouses of male alcoholic patients | 46 | Bharatpur, Nepal | NA | Depressive Disorder, Conversion Disorder, Anxiety Disorder, Somatoform Disorder, Bipolar Disorder,Psycho Sexual Disorder | NA | NA |
| 43 | Sharon P., 2014 | Hospital setting | Ex-Post Facto Research Design | To compare two groups namely wives of alcoholics (Sample 1- Study group) with a comparative group of wives of non-alcoholics (Sample 2- Control Group | 300 | Tiruchirappalli, Tamil Nadu, India | Their husbands physically abused the family  members. | Verbally abused the family members and got into quarrels with them. Low level of perceived quality of life | NA | NA |
| 44 | Shiji., 2020 | Community setting | Descriptive survey research design | Total 150 men residing at Mangalore and Bantwal taluk of Dakshina Kannada district were administered the AUDIT tool through house to house survey, of which 132 men who scored 8 to 10 were taken as alcoholics and their wives were selected as participants through purposive sampling technique. | 150 | Mangalore, Karnataka, India | NA | Poor quality of life experienced by wives whose husbands are alcoholics. (physical domain, psychological domain, social domain and environmental domain). | NA | NA |
| 45 | Sidhu et al., 2016 | Tertiary care teaching hospital setting | Cross-sectional observational study | 25 children aged between 6 and 18 years, having no known psychiatric illness were interviewed. Their parent was diagnosed as per ICD-10 as alcohol dependence syndrome. The other parent who was not alcohol dependent was assessed using a general health questionnaire (GHQ-28) scale. If the score of this parent was <24 on GHQ-28, the child was considered as a part of the study. | 7 | Maharashtra, India | NA | Affective(girls>boys) and anxiety(boys>girls) problems amongst COAs, ADHD(boys>girls) and conduct (boys>girls) problems amongst COAs, Somatic (only girls)and ODD(boys>girls) problems amongst COAs. The girls had more internalizing problems as compared to boys who had more externalizing problems. Dysfunction in all the three dimensions, namely the relationship, personal growth and the system maintenance. | NA | NA |
| 46 | Stanley S., 2008 | De-addiction facility run by a NGO | Cross sectional ex-post facto research design. | 75 wives of alcoholics with an equal number of wives of non-alcoholics matched. This comparative study draws on two respondent populations, one clinical, and the other from the community. It is based on the presumption that the effect if any, of living with an alcoholic (study group) or non-alcoholic (reference group) spouse would have already manifested itself on the marital experience of both groups. | 150 | Tiruchirappalli, Tamil Nadu, India | NA | Higher levels of conflict, experience greater communication apprehension, and perceive a higher threat of danger in their marital relationship. | NA | NA |
| 47 | Subodh NB et al., 2014 | De-addiction centre, Hospital in PGIMER | Cross-sectional study | Of the 380 patients, 267 participated in the study along with their wives. | 534 | Chandigarh, India | Prevalence of IPV. Comparatively, the alcohol group reported higher mean number of physical violence. Different types of specific violent behaviours, like being grabbed or shoved, punched on the body/arms/legs, punched in the face, attempt to burn, criticism and locked in the house, Forced you to have sex, Physically violent to you in other way, Kicked you on the floor, Choked or held hand over your mouth were reported more by the alcohol group. | Criticism | NA | NA |
